# The application of spectroscopy techniques for diagnosis of malaria parasites and arboviruses and surveillance of mosquito vectors: A systematic review and critical appraisal of evidence

**DOI:** 10.1371/journal.pntd.0009218

**Published:** 2021-04-22

**Authors:** Brendon Goh, Koek Ching, Ricardo J. Soares Magalhães, Silvia Ciocchetta, Michael D. Edstein, Rafael Maciel-de-Freitas, Maggy T. Sikulu-Lord

**Affiliations:** 1 School of Public Health, Faculty of Medicine, The University of Queensland, Brisbane, Australia; 2 UQ Spatial Epidemiology Laboratory, School of Veterinary Science, The University of Queensland, Brisbane, Australia; 3 Children’s Health Research Centre, Children’s Health and Environment Program, The University of Queensland, Brisbane, Australia; 4 Australian Defence Force, Malaria and Infectious Disease Institute, Brisbane, Australia; 5 Laboratório de Transmissores de Hematozoários, Instituto Oswaldo Cruz, Rio de Janeiro, Brazil; University of Glasgow, UNITED KINGDOM

## Abstract

Spectroscopy-based techniques are emerging diagnostic and surveillance tools for mosquito-borne diseases. This review has consolidated and summarised recent research in the application of Raman and infrared spectroscopy techniques including near- and mid-infrared spectroscopy for malaria and arboviruses, identified knowledge gaps, and recommended future research directions. Full-length peer**-**reviewed journal articles related to the application of Raman and infrared (near- and mid-infrared) spectroscopy for malaria and arboviruses were systematically searched in PUBMED, MEDILINE, and Web of Science databases using the PRISMA guidelines. In text review of identified studies included the methodology of spectroscopy technique used, data analysis applied, wavelengths used, and key findings for diagnosis of malaria and arboviruses and surveillance of mosquito vectors. A total of 58 studies met the inclusion criteria for our systematic literature search. Although there was an increased application of Raman and infrared spectroscopy-based techniques in the last 10 years, our review indicates that Raman spectroscopy (RS) technique has been applied exclusively for the diagnosis of malaria and arboviruses. The mid-infrared spectroscopy (MIRS) technique has been assessed for the diagnosis of malaria parasites in human blood and as a surveillance tool for malaria vectors, whereas the near-infrared spectroscopy (NIRS) technique has almost exclusively been applied as a surveillance tool for malaria and arbovirus vectors.

**Conclusions/Significance:**

The potential of RS as a surveillance tool for malaria and arbovirus vectors and MIRS for the diagnosis and surveillance of arboviruses is yet to be assessed. NIRS capacity as a surveillance tool for malaria and arbovirus vectors should be validated under field conditions, and its potential as a diagnostic tool for malaria and arboviruses needs to be evaluated. It is recommended that all 3 techniques evaluated simultaneously using multiple machine learning techniques in multiple epidemiological settings to determine the most accurate technique for each application. Prior to their field application, a standardised protocol for spectra collection and data analysis should be developed. This will harmonise their application in multiple field settings allowing easy and faster integration into existing disease control platforms. Ultimately, development of rapid and cost-effective point-of-care diagnostic tools for malaria and arboviruses based on spectroscopy techniques may help combat current and future outbreaks of these infectious diseases.

## Author summary

Malaria and many arboviruses such as Dengue virus, Zika virus, Chikungunya virus, and Ross River virus are persistent and detrimental to the global population. Rapid and accurate diagnosis of these infections in human populations and mosquito vectors is essential for understanding their epidemiology, for prompt treatment, and to improve and guide control and elimination strategies. Raman and infrared spectroscopy are rapid and cost-effective tools that have shown potential as diagnostic and surveillance tools for malaria and arboviruses. This systematic review presents up-to-date research conducted using RS, MIRS, and NIRS for the diagnosis of malaria parasite and arboviruses as well as for the surveillance of malaria and arbovirus vectors.

## Introduction

Malaria is a mosquito-borne disease caused by the *Plasmodium* parasite and transmitted to humans and other animals through the bite of an infected female *Anopheles* mosquito [1]. In 2019, an estimated 229 million malaria cases and 409,000 malaria-related deaths were reported, highlighting malaria as a major public health concern [2]. Arboviruses such as Chikungunya (CHIKV), Dengue (DENV), and Zika (ZIKV) are transmitted to humans through bites of infected *Aedes* mosquitoes. CHIKV cases have been reported in Africa, Asia, Americas, and Europe causing an estimated 693,000 annual cases and an epidemic in over 50 countries [3,4]. The risk of death with CHIKV is approximately 1 in a 1,000 [5]. DENV infections have increased dramatically over the last 20 years, particularly in tropical countries. It is estimated that at least 390 million infections occur each year of which 96 million manifests clinically [6]. ZIKV caused an epidemic in Brazil between 2015 and 2016 resulting in approximately 1.6 million infections and 5,968 cases of microcephaly in newborns [7].

### Diagnosis of malaria and arboviruses

To achieve the aims set by the World Health Organisation’s (WHO) Global Technical Strategy for Malaria 2016–2030 which aims to reduce malaria incidence and related mortality by 90% and to eradicate malaria in at least 35 countries by 2030, new strategies to address residual malaria transmission and tools to monitor the results of these strategies are urgently needed [8]. One of the cornerstones for disease control is the availability of good quality vaccines; however, malaria and some arboviruses vaccines are still under development. For example, the only approved malaria vaccine RTS,S (Mosquirix) has a relatively low efficacy and is not recommended by WHO for vaccination of babies between 6 to 12 weeks of age [9]. To achieve the WHO’s 2030 goals of reducing malaria-related mortality by 90%, diagnosis of malaria and mosquito surveillance have been pinpointed as fundamental tools [8]. In addition, to reduce the spread and unprecedented future outbreaks of mosquito-borne diseases, active surveillance of vectors and parasites within human populations is crucial.

Current diagnosis of malaria relies primarily on microscopy methods using Giemsa stained blood smears [10]. However, with a limit of detection of >5 parasites/μL of blood, it requires a well-trained microscopist [11]. Rapid diagnostic tests are also common diagnostic tools for malaria. They are very easy to use and do not require qualified personnel, but their sensitivity and specificity is low in detecting low parasitaemia [12]. Molecular based techniques such as polymerase chain reaction (PCR), quantitative PCR (qPCR), nested PCR, and enzyme-linked immunosorbent assay (ELISA) have also been developed for malaria [13] and arboviruses [14,15]. PCR techniques are gold standards for the diagnosis of arboviruses; however, due to time, cost inefficiencies, and technical expertise required, they are unsuited for large-scale diagnoses particularly during disease outbreaks. For example, the cost of DENV1 antibody ELISA kit is approximately $10.4 USD per sample [16], while a malaria IgG and IgM antibody ELISA kit costs are estimated at $5.5 USD per sample [17]. Additionally, basic laboratory skills are required to perform PCR or ELISA techniques efficiently and correctly.

### Vector surveillance of malaria and arboviruses

Vector surveillance involves regular monitoring of mosquito populations to assess the effectiveness of vector control interventions. Surveillance assesses vector survival (age), species diversity, infection status, host preference, and insecticide resistance. These parameters are currently determined using molecular techniques including PCR and qPCR or ELISA [18–20]. Vector survival is the most important determinant of vectorial capacity of mosquito vectors. Mosquito age prediction can be useful in identifying potentially infectious vectors, as pathogens must incubate for a certain period of time within mosquitoes before they can be transmitted to hosts. For example, the female *Anopheline* mosquito can only transmit *Plasmodium* parasites after 10 to 12 days following ingestion of an infected blood meal due to the long incubation period required for *Plasmodium* parasite development within the vector [21]. Consequently, a mosquito population that survives longer that this extrinsic incubation period will be more likely to transmit malaria to susceptible hosts.

Parity dissections to determine whether a mosquito has previously laid eggs or not is the current gold standard technique to determine mosquito age [22]. A related technique which determines the number of dilatations in the ovaries indicates how many times a mosquito has laid eggs [23]. Although these techniques require minimal reagents to operate, they are time consuming and tedious allowing only a small proportion of samples to be dissected at a time which can be an accurate representation of the age composition of a mosquito population.

### Raman and infrared spectroscopy

Raman spectroscopy (RS) is a technique that provides chemical fingerprints of molecules by determining their vibrational modes through inelastic scattering of photons known as Raman scattering or Raman effect [24]. During Raman scattering, molecules gain energy from an incident light source. Raman effect is therefore the difference between monochromatic incident and exit radiation. One of the important features that makes RS useful in biological applications is its ability to avoid interference by water molecules. This is due to inability of water to induce Raman scattering. RS can be used as a quantification and identification measure for biological samples. For example, RS has been used to identify molecular compositions in biological samples such as the eyes [25], teeth [26], muscles [27], and nerves [28] and quantify molecular compositions in blood [29–31].

**Fig 1:**
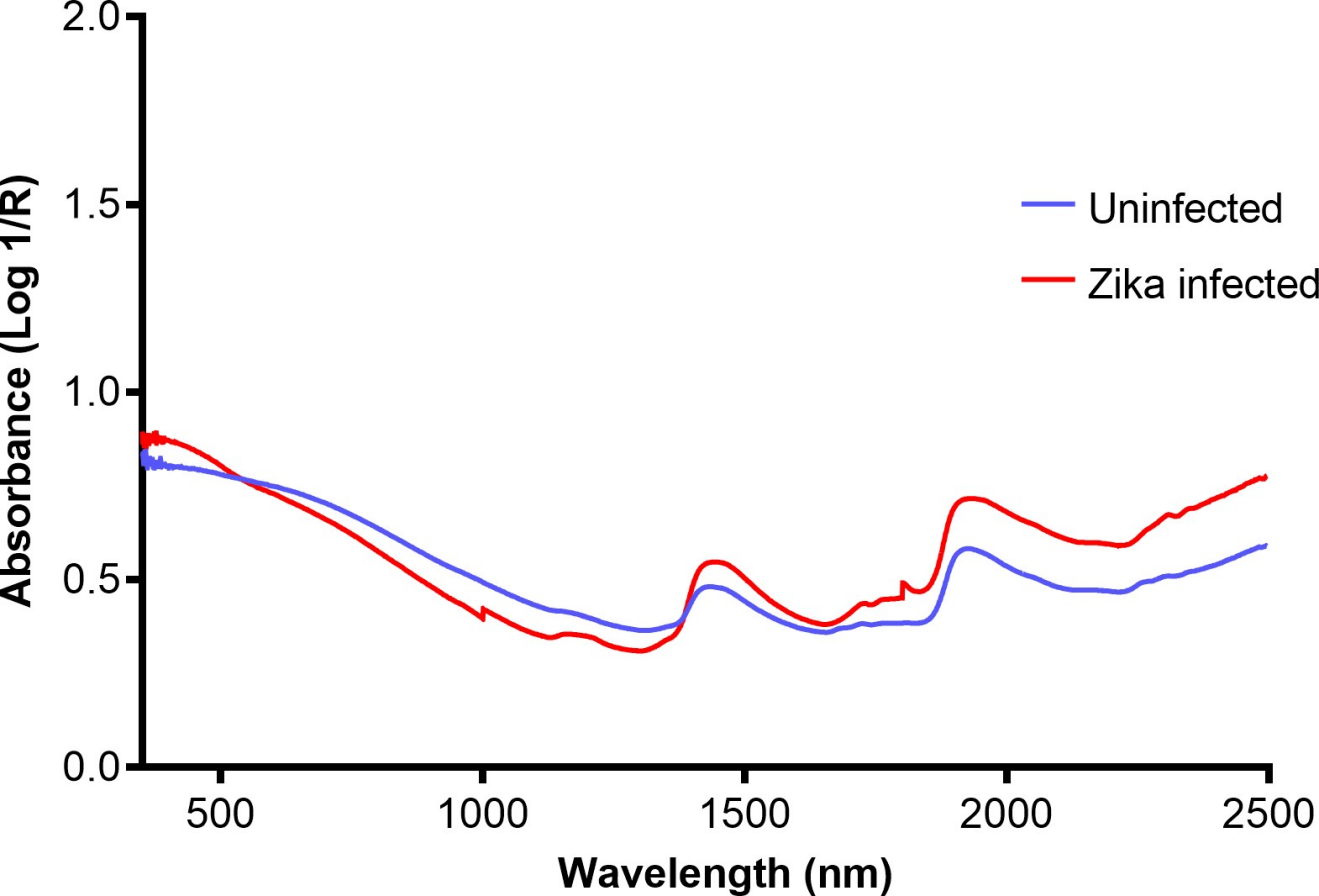
An example of an averaged NIRS raw spectra collected from the heads and thoraces of ZIKV-infected (red) and uninfected (blue) *Ae*. *aegypti* mosquitoes. Adapted from Fernandes and colleagues [90].

**Fig 2:**
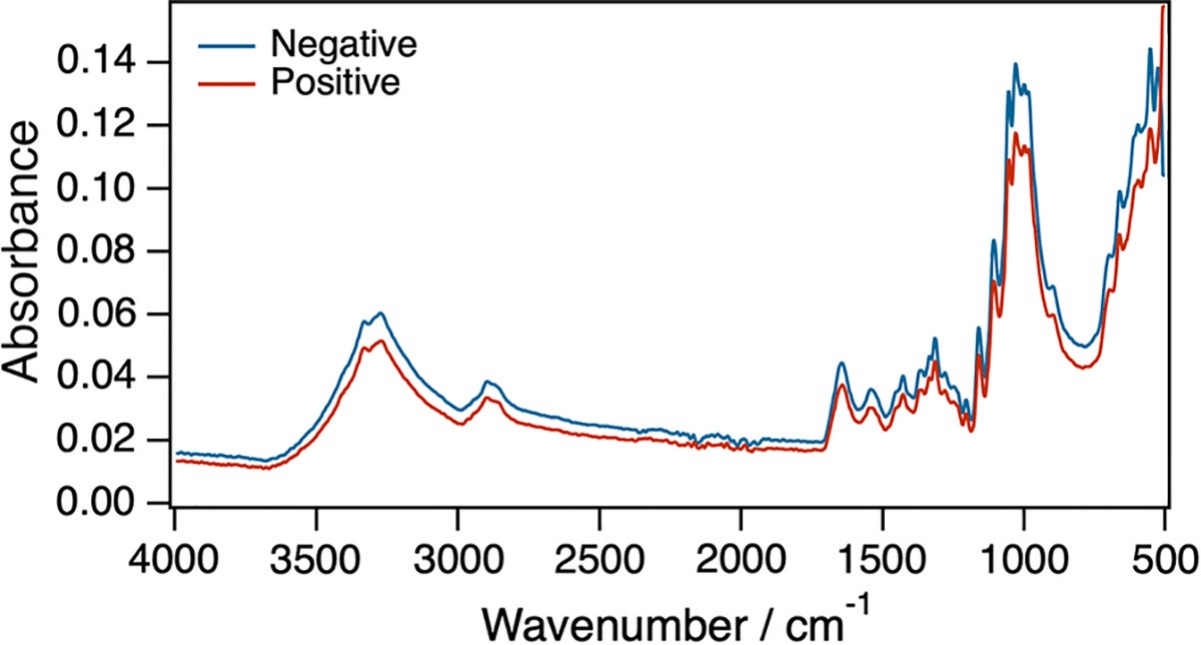
An example of an averaged MIRS spectra from dried blood infected with malaria parasites (red spectrum) and uninfected blood (blue spectrum). Adapted from Mwanga and colleagues [70].

Infrared spectroscopy involves the interaction of infrared radiation with biological samples to produce a diagnostic spectrum. It capitalises on the fact that molecules absorb light at specific frequencies characteristic of their chemical composition [32]. This implies that different biological samples with varying chemical profiles have unique absorption and reflectance properties characteristic of their functional groups and can therefore be quantified as peaks on an infrared spectrum. The infrared portion of the electromagnetic spectrum consist of 3 regions: near-, mid-, and far-infrared. The near-infrared region consists of frequencies that range from 14,000 to 4,000 cm^−1^ (800 to 2,500 nm wavelength) and is generally used to observe excitation of overtone or harmonic molecular vibrations (Fig 1). The mid-infrared region consists of frequencies that range from 4,000 to 400 cm^−1^ (2,500 to 25,000 nm wavelength) and is used to study key rotational-vibrational structure (Fig 2). Therefore, mid-infrared wavelengths provide more detailed analyses of a sample. Unlike near-infrared, mid-infrared is invasive and is unsuited for in vivo studies.

Both Raman and infrared spectroscopy techniques are rapid and inexpensive techniques compared to molecular and microscopy techniques for similar purposes. Although the initial outlay for a NIR spectrometer can be costly (approximately $40,000 USD), benefits such as minimal sample processing, large-scale applications, and minimal labour can outweigh these initial cost in the long run [33].

Chemometrics/machine learning techniques are usually coupled with spectroscopy techniques to produce diagnostic information required for sample characterisation. Following development of training models, these techniques only require basic computer and spectra collection skills. However, to date, there has not been a comprehensive review of applications of these spectroscopy techniques for mosquito-borne diseases.

We systematically reviewed published evidence from 2009 to 2021 involving the use of Raman and infrared spectroscopy techniques for the diagnosis of malaria parasites and arboviruses and for surveillance of mosquito vectors.

## Methods

### Search strategy

Standard systematic review and meta-analysis (PRISMA) guidelines were applied for this review [34]. We searched PUBMED, MEDILINE, and Web of Science databases for peer-reviewed journal articles published from 2009 to 2021 (January). We manually searched reference lists of included articles to capture relevant articles [35]. To identify articles on the application of RS in the field of mosquito-borne diseases, the following key terms were used: “Raman spectroscopy arboviruses,” “Raman spectroscopy malaria,” “Raman spectroscopy Chikungunya,” “Raman spectroscopy Dengue,” “Raman Spectroscopy mosquitoes,” “Raman spectroscopy Ross River,” and “Raman spectroscopy Zika.” The application of infrared techniques was searched in the same databases with the following key terms: “Infrared spectroscopy arbovirus,” “Infrared spectroscopy malaria,” “Infrared spectroscopy Chikungunya,” “Infrared spectroscopy Dengue,” “Infrared spectroscopy mosquitoes,” “Infrared spectroscopy Ross River,” and “Infrared spectroscopy Zika.” No restrictions were applied to language. EndNote software (Thompson Reuters, Philadelphia, Pennsylvania, United States of America) was used to store articles retrieved from databases which were screened for duplicates. Titles and abstracts were screened by two authors (BG and KC) to identify relevant publications that met the inclusion criteria. Full-text review was applied by one author (BG) to determine the eligibility of articles. Eligible articles were grouped into 3 categories based on the spectroscopy technique used, RS, MIRS, and NIRS.

### Inclusion and exclusion criteria

**Fig 3:**
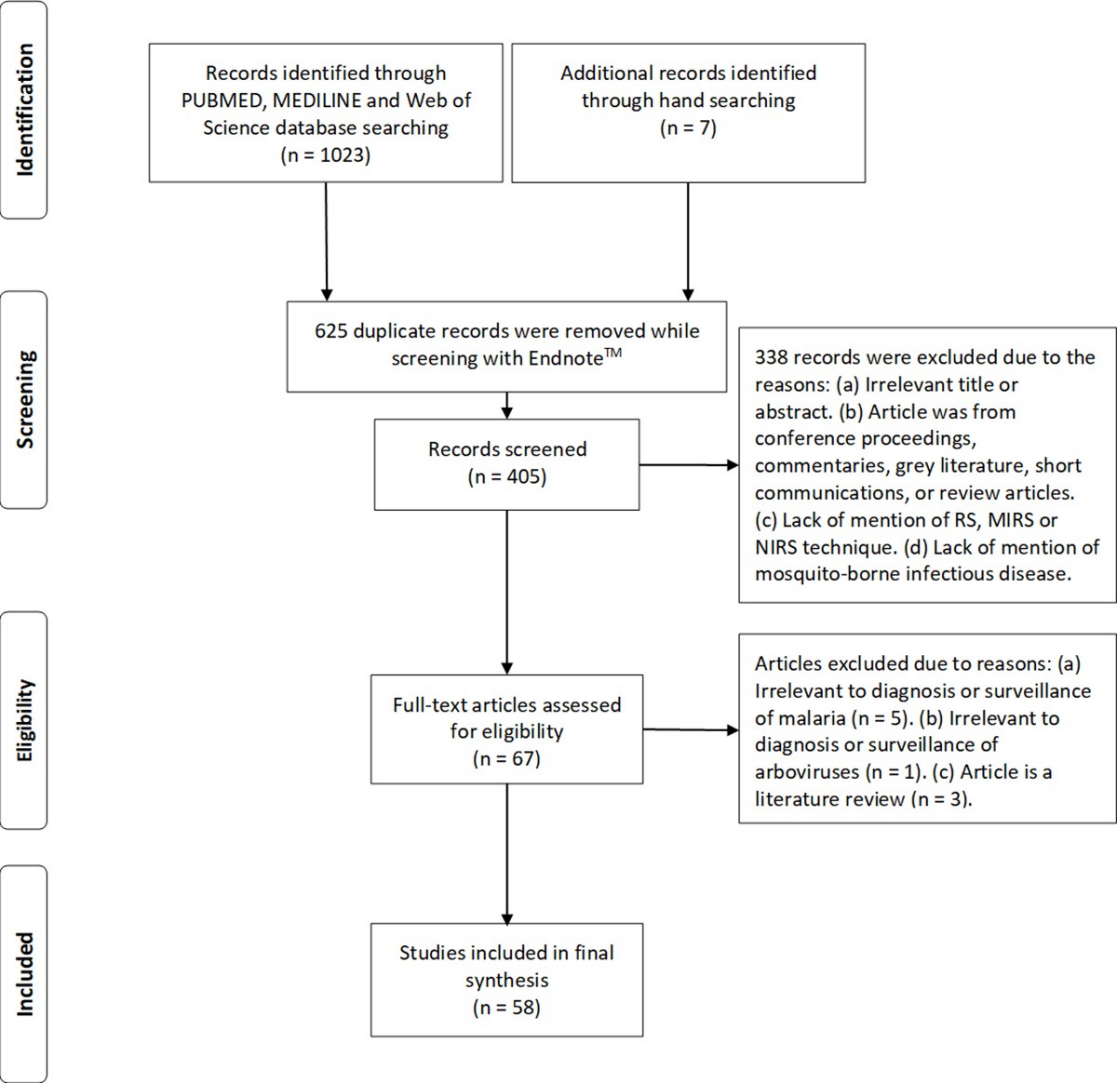
Search and selection process based on PRISMA framework. A total of 58 peer-reviewed articles from 2009 to 2021 (January) were reviewed.

Articles were eligible for inclusion if they demonstrated use of RS, MIRS, and NIRS for the diagnosis, detection, and visualisation of malaria parasites, arboviruses, or surveillance of mosquito vectors. Machine learning articles involving the same sample types were also included in this review. Articles were excluded based on the following criteria: (a) abstract or full paper was not accessible; (b) article does not mention RS, MIRS, or NIRS techniques; (c) article does not mention mosquito-borne diseases; (d) RS, MIRS, or NIRS technique was not used in the main experiments; and (e) conference proceedings, commentaries, grey literature, short communications, or review articles (Fig 3). Articles excluded are indicated in S2 Table.

### Data extraction

Eligible articles were subjected to data extraction based on the following criteria: (a) type of spectroscopy technique used; (b) type of sample analysed; (c) method of sample preparation; (d) method of sample analysis; (e) method of data analysis; and (f) result of the experiment based on the spectroscopy technique used (i.e., accuracy defined as the percentage of correct predictions for a sample set, sensitivity defined as the proportion of positive samples that are correctly predicted as positive, and specificity defined as the proportion of negative samples that are predicted as negative).

### Results of search strategy

#### Characteristics of journal articles included in this systematic review

A total of 1,023 peer-reviewed journal articles were identified through PUBMED, MEDILINE, and Web of Science database searches. Seven peer-reviewed journal articles were identified by hand searching eligible articles. A total of 405 unique articles were retained after duplicates were removed using Endnote software. A total of 67 articles met our inclusion criteria and were subjected to full text review. After full text assessment, 58 articles met our inclusion criteria for this systematic review (Fig 3).

#### Time trend of journal articles in this systematic review

**Fig 4:**
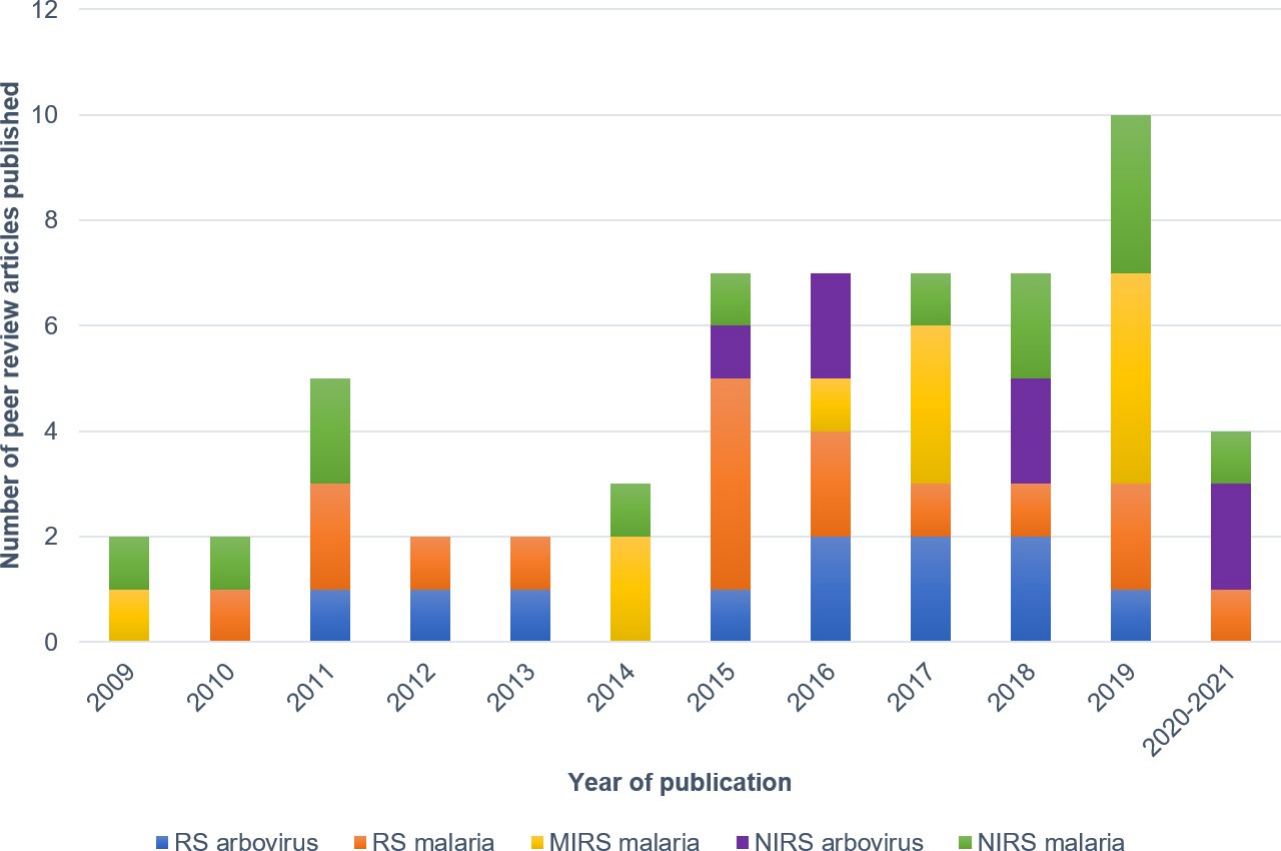
The number of articles included in this literature review that are related to RS, MIRS, and NIRS for the diagnosis and surveillance of malaria and arboviruses classified by year of publication.

There has been an upward trend in the number of peer-reviewed articles published in the field of RS, NIRS, and MIRS from 2009 to 2019, with a more than 2-fold increase in 2015. In 2011, a spike in the number of NIRS and RS articles published was observed. A further increase in articles was observed in 2019 which was mainly associated with the application of MIRS in the field of malaria. A decline in studies from 2020 onwards is likely due to the ongoing COVID-19 pandemic (Fig 4).

In this review, 27 RS, 11 MIRS, and 20 NIRS studies were included. RS, MIRS, and NIRS studies were first split into 2 groups; malaria and arbovirus studies and each of the 2 groups was further split into articles that focused on diagnostics and vector surveillance (Table 1). All reviewed articles under RS were related to its application for the diagnosis of malaria or arboviruses in whole blood/red blood cells (RBCs)/serum/serum. All MIRS articles were related to its use for the diagnosis of malaria parasite in RBCs and surveillance of malaria vectors. Finally, all but one NIRS article reviewed assessed its use as a vector surveillance tool for malaria and arbovirus vectors.

**Table 1:** Summary of all articles included in this literature review sorted by type of spectroscopy method and scope of study.

Scope of study	Spectroscopy Technique		
	RS	MIRS	NIRS
Malaria parasite diagnostics	16	8	1
Malaria vector surveillance	0	3	12
Arbovirus diagnostics	11	0	0
Arbovirus vector surveillance	0	0	7
**Total number of studies**	**27**	**11**	**20**

MIRS, mid-infrared spectroscopy; NIRS, near-infrared spectroscopy; RS, Raman spectroscopy.

## Results and discussion

### Application of RS for the diagnosis of malaria parasites

The application of RS to differentiate ring, trophozoite, and schizont stages of the malaria parasite in human O+ RBCs has been demonstrated. *Plasmodium falciparum-*infected RBCs at the ring stage showed a characteristic Raman peak at 6,254 nm, while trophozoite and schizont stages had distinct peaks at 13,831 nm. The difference in the characteristic peaks were attributed to the modification of the RBC membrane during the development of the parasite [36]. Another strategy to enhance limit of detection of RS resonance utilised silver nanoparticles synthesised within *P*. *falciparum* parasites. A limit of detection of 2.5 parasites/μL for the ring stage of *P*. *falciparum* was achieved [37]. In a separate study, both malaria and DENV patient’s whole blood samples were differentiated from healthy samples with 83.3% accuracy with positive likelihood ratios of 0.9529 and 0.9584, respectively [31]. When RS was used to predict *Plasmodium vivax* infection in human plasma, an accuracy of 86% was achieved [38], whereas 1 parasite/μL of either *P*. *falciparum* and *P*. *vivax* infections in whole blood could be detected with surface-enhanced Raman spectroscopy (SERS) coupled with a nanostructured gold substrate [39]. A study on how pressure affects the Raman spectra was carried out on synthetic hematin anhydride equivalent to malaria pigment hemozoin. The intensity of RS peaks decreased when synthetic hematin anhydride was subjected to increasing pressure up to 27 kbar above atmospheric pressure [40]. As malaria is more prevalent in tropical countries, therefore, higher temperatures in those countries could lead to increased pressure within blood samples during long-term storage. This can influence the RS diagnostic signature obtained. Further tests using real-world malaria-infected blood samples are required to confirm this phenomenon.

RS has also been used to study both blood and tissue stages of *Plasmodium berghei*-infected mice. In a study reported by Hobro and colleagues [41], *P*. *berghei* infection progression was monitored with a confocal Raman microscope via infected mouse at days 1, 2, 3, 4, and 7 post inoculation. Heme-based changes were observed in mice at a parasitaemia of 0.2% in plasma, and erythrocyte membrane changes were observed on day 4 post inoculation at 3% parasitaemia [41]. In mice infected with the *P*. *berghei* ANKA strain, significantly higher heme-based Raman vibrations were observed in the tissue of mice with 5% parasitaemia compared with tissues of uninfected mice indicating possible presence of hemozoin [42].

Several other studies applied RS to visualize the hemozoin pigment produced during malaria infection. An atomic force Raman microscope was used to observe the effect of in vitro treatment procedures on *P*. *falciparum*-infected RBCs. Results showed that infected RBCs dried in phosphate buffer solution (PBS) causes localisation of hemichrome at the periphery of RBCs, formaldehyde causes diffusion of haemoglobin into the surrounding areas of the RBCs, while a mixture of formaldehyde (3%) and glutaraldehyde (0.1%) maintained the structural integrity of RBCs [29]. Other studies involving RS visualisation of malaria infection in RBCs include studies that show (a) an increase in hemozoin crystal size over time of infection [43], (b) improvement in visualization of β-hematin crystals with the use of magnetised iron oxide core and silver shell nanoparticles [44], (c) similarity in biochemical compounds found in intracellular and extracellular hemozoin [45], (d) an increase in intravascular heme solubility due to nitric oxide interaction with heme [46], (e) a reduced oxygen-affinity for intracellular haemoglobin [30], and (f) identification of five-coordinate high-spin ferric heme complex in erythrocyte digestive vacuole [47].

Structural analysis of *P*. *falciparum*-infected RBCs using RS indicated a lower number of domains arranged in transconformation, an increase in membrane protein and lipids, an increase in deoxygenated haemoglobin, and a decrease in α-helical content with an increase in undefined structures [48]. When resonance RS was used for the structural analysis of iron porphyrins and β-hematin, solid states iron porphyrin [Fe(OEP)]_2_O exhibited total symmetric mode *v*_4_ when excited with 782 nm and 830 nm lasers. It was also observed that less supramolecular interactions were present. Based on the difference in excitation and supramolecular interactions, the authors suggested that the intensity of symmetric mode *v*_4_ is strongly affected by C–H–X hydrogen bond interactions [49]. A summary of the studies that applied RS for diagnosis of malaria are shown in Table 2.

**Table 2:** A summary of articles reviewed that applied RS for the diagnosis of malaria parasite.

Objective/s	Key finding/s	Excitation wavelength/nm	Data processing technique	Reference
To study the effects of in vitro treatment procedures on human RBCs infected with *P*. *falciparum* with atomic force Raman microscope and Raman mapping.	Micro-Raman maps and atomic force Raman microscopic images were related to heme content in fixed and dried RBCs. Hemichrome was found at periphery of cells when RBCs were dried in PBS. Formaldehyde as a fixative causes diffusion of haemoglobin into the surrounding area of RBCs while a mixture of formaldehyde (3%) and glutaraldehyde (0.1%) retained the structural integrity of RBCs with minimal autofluorescence allowing *P*. *falciparum*-infected RBCs to retain knob like structures.	532	Unsupervised hierarchical cluster analysis	[29]
To differentiate *P*. *vivax* infected from uninfected human RBCs with fiber array-based hyperspectral Raman imaging using Raman optical tweezers.	Raman spectra peaks at regions 1,210–1,223, 1,356–1,366, and 1,544–1,636 cm^−1^ were responsible for differentiating healthy from *P*. *vivax*-infected RBCs.	785	None	[30]
To monitor nitric oxide coordination with human RBCs heme in isolated food vacuoles of *P*. *falciparum* using Resonance RS.	Nitric oxide interacts with heme in food vacuoles to form ferrous heme nitrosyl complexes which influence intravascular heme solubility.	406.7	None	[46]
To observe *P*. *falciparum* hemozoin crystals in sectioned human RBCs with tip enhanced atomic force Raman microscope.	RBCs were fixed in glutaraldehyde (0.1%) and formaldehyde (2%) and embedded in LR-white medium. Crystals with five-coordinate high-spin ferric heme complex were observed in a digestive vacuole of a malaria parasite infected cell.	532	PCA	[47]
To improve identification of β-hematin crystals with magnetic field enriched SERS.	β-hematin detection limit was approximately 30 parasites/μL with iron oxide core and silver shell nanoparticles. Signal intensities were improved by 3 and 5 orders of magnitude with nanoparticles and when nanoparticles were magnetised, respectively.	633	None	[44]
To monitor *P*. *berghei* infection progression in mice blood and plasma with confocal Raman microscope.	Heme-based changes could be detected at 0.2% parasitaemia in plasma and erythrocyte membrane changes were observed when parasitaemia levels reached 3%.	532	PCA	[41]
To diagnose *P*. *vivax* in human plasma with resonance RS.	R^2^ value of 0.981 for training models were obtained. Independent validation set yielded 86% accuracy.	532	PLS	[39]
To analyse *P*. *falciparum*-infected human RBCs with fiber array-based hyperspectral Raman imaging.	Hemozoin deposits in *P*. *falciparum*-infected RBCs and differences based on infection time (up to 44 hours) was observed. Hemozoin crystals increased in size over time.	532	None	[43]
To investigate biochemical changes occurring in macrophages during hemozoin uptake using confocal Raman microscope.	Regardless of the macrophage location, intracellular and extracellular hemozoin were biochemically similar. Some hemozoin were associated with lipid-based components. Spatial distribution of hemozoin was observed to be inhomogeneous.	532	Singular value decomposition and PCA	[45]
To analyse the effect of ring-stage *P*. *falciparum* in human RBCs with confocal Raman microscope.	Raman spectra differences between infected and healthy human RBCs indicated that there was a lower number of domains which were arranged in transconformation, an increase in membrane protein and lipids, an increase in deoxygenated haemoglobin and a decrease in α-helical content with concurrent increase in undefined structures in *P*. *falciparum*-infected RBCs.	785	PCA	[48]
To detect and differentiate *P*. *falciparum* ring stage from trophozoite and schizont stages in infected human RBCs with SERS.	*P*. *falciparum* at ring stage had characteristic Raman peak at 1,599 cm^−1^ while trophozoite and schizont stages were both detected at 723 cm^−1^.	785	PCA	[36]
To detect hemozoin in *P*. *falciparum*-infected human blood using SERS with silver nanoparticles synthesised inside or outside lysed RBCs.	Limit of detection was 2.5 parasites/μL when nanoparticles were synthesised internally and 500 parasites/μL when synthesised externally.	633 nm	PLS	[37]
To identify differences in *P*. *berghei* ANKA infected and noninfected tissue with confocal Raman microscope.	Although imaging data indicated similar biochemical profiles for the infected and uninfected tissues, the presence of heme-based Raman vibrations in infected cells indicated presence of hemozoin.	532	PCA	[42]
To investigate the effects of pressure on synthetic β-hematin spectra with resonance RS.	Raman shift for key Raman active bands (V_2_, V_10_, V_15_, V_30_, V_37_, V_40_, and V_42_) and a decrease in intensity of Raman bands (at 718 nm excitation) was observed when pressure was increased to 27 kbar (relative to atmospheric pressure). Changes in chemical bond energies were identified to be due to Fe (III) vibrations.	514, 633, and 718	None	[40]
To rapidly distinguish between malaria-infected, DENV-infected, and healthy patient’s blood plasma with resonance RS^a^.	Raman spectra accuracy for malaria and DENV distinction was 83.3%. Malaria infected vs healthy controls and DENV infected vs healthy controls had positive likelihood ratios of 0.9529 and 0.9584, respectively.	785	PCA-DA	[31]
To identify *P*. *falciparum* and *P*. *vivax* infection in whole blood with nanostructured gold substrate SERS.	Peaks at 1,370, 1,570, and 1,627 cm^−1^ were associated with *P*. *falciparum* and *P*. *vivax* infection. Limit of detection for *P*. *falciparum*-parasitised blood cells was 1 parasite/μL.	532	None	[38]

^a^In addition to malaria, this study also presents findings on the application of RS for diagnosis of DENV.

DENV, Dengue virus; PCA, principal component analysis; PCA-DA, principal component analysis-factorial discriminant analysis; PLS, partial least squares; RBC, red blood cell; RS, Raman spectroscopy; SERS, surface-enhanced Raman spectroscopy.

### Application of RS for the diagnosis of arboviruses

A sensitivity of 97.38% and a specificity of 86.18% were achieved when RS was used to detect DENV in blood plasma relative to ELISA testing for nonstructural protein 1, immunoglobulin M (IgM), and immunoglobulin G (IgG) [50]. However, a lower predictive accuracy of 66% and 47% was observed when RS was used to detect DENV-infected blood sera relative to IgG and IgM ELISA tests, respectively. The predictive accuracy of DENV based on IgG antibodies was lower than the accuracy based on IgM probably because IgM is generated at the onset of the infection before IgG. Overall, low accuracies were due to high false negative results [51]. Elevated lactate concentration in human blood sera was observed in DENV-infected patients possibly due to impaired function of body organs [52]. When support vector machine (SVM) learning models coupled with polynomial kernel order 1 were applied, DENV was predicted with a predictive accuracy of 85% and a sensitivity of 73% [53]. In a recent study, RS was used to differentiate between bacteria (*Salmonella Typhi*) and virus (DENV) infections in human blood serum, with 12 distinct Raman bands linked to typhoid-infected samples and 4 to DENV [54].

Multiple studies have been conducted to determine the limit of detection of RS for identifying arbovirus-related antigens, with emphasis on Rift Valley fever virus (RVFV) and West Nile virus (WNV) using SERS coupled with gold and silver nanotags [55–58]. When gold paramagnetic nanoparticles were used, the limit of detection for WNV-specific target DNA sequence was 10 pM [57]. The same methodology was used to identify the limit of detection of RVFV (20 nM) and WNV (100 nM) based on their RNA sequences [58]. RS and gold paramagnetic nanoparticles tags were also used to simultaneously detect WNV and RVFV where detection limits of 5 fg/mL and approximately 25 pg/mL were achieved when viruses were suspended in PBS or PBS with fetal bovine serum, respectively [56]. However, when a polyacrylic acid layer was applied, a reduction in background noise was observed and a limit of detection of 10 pg/mL for WNV, RVFV, and *Yersinia pestis* antigens was achieved [55].

Detection limits as low as 10 plaque-forming units (PFU)/mL were achieved for both DENV and WNV when SERS coupled with a bioconjugated gold nanoparticle was used [59]. Whereas a limit of detection of 7.67 ng/mL for DENV and 0.72 ng/mL for ZIKV was achieved by coupling RS to a lateral flow assay [60]. These detection limits are lower relative to ELISA detection limits of 120 ng/mL for ZIKV, <1 ng/mL for DENV, and 61 PFU/mL for WNV [61–63]. A summary of the studies that applied RS for the diagnosis of arboviruses are shown in Table 3.

**Table 3:** A summary of the articles reviewed that applied RS for the diagnosis of arboviruses.

Objective/s	Key finding/s	Excitation wavelength/nm	Data processing technique	Reference
To detect WNV DNA with SERS and magnetic capture Raman active gold nanoparticles.	The limit of detection for the target sequence was 10 pM.	785	None	[57]
To detect RVFV and WNV DNA with SERS and silver-coated paramagnetic nanoparticles.	The limit of detection for RVFV and WNV was 20 nM and 100 nM, respectively.	785	None	[58]
To detect RVFV N and WNV E proteins with SERS and magnetic capture nanoparticles.	For both viruses, the limit of detection was approximately 5 fg/mL in PBS and approximately 25 pg/mL in PBS with fetal bovine serum.	785	None	[56]
To detect DENV2 and WNV (from in vitro cells) with bioconjugated gold nanoparticle-based SERS.	The limit of detection for DENV2 and WNV particles was 10 PFU/mL.	670	None	[59]
To classify DENV infected from healthy human blood sera with resonance RS.	An accuracy, precision, sensitivity and specificity of 85%, 90%, 73%, and 93%, respectively, was obtained.	532	SVM	[53]
To identify DENV-infected patients blood sera with resonance RS in comparison to ELISA for detecting IgG and IgM.	Relative to IgG, an accuracy, precision, specificity, and sensitivity of 66%, 70%, 72%, and 61% was achieved, respectively. Relative to IgM, an accuracy, precision, specificity, and sensitivity of 47%, 46%, 52%, and 43% was achieved, respectively.	532	None	[51]
To screen for DENV in human blood sera via observation of lactate concentration with resonance RS.	An increased intensity at 750, 830, and 1,450 cm^−1^ and decreased intensity at 1,003, 1,156, and 1,516 cm^−1^ were observed for DENV-infected blood sera samples relative to controls.	532	None	[52]
To distinguish between ZIKV and DENV NS1 biomarkers with SERS combined with a lateral flow assay.	Detection limit for ZIKV NS1 was 0.72 ng/mL and the detection limit for DENV NS1 was 7.67 ng/mL.	785	PLS	[60]
To identify DENV-infected patients blood sera with resonance RS in comparison to ELISA for detecting NS1 protein, IgG, and IgM.	A sensitivity of 97.38% and a specificity of 86.18% was achieved when RS was evaluated against NS1 protein, IgM, and IgG ELISA.	785	PCA-DA	[50]
To detect WNV, RVFV, and *Yersinia pestis* in fetal bovine serum with SERS using silica-encapsulated nanotags.	Limit of detection was approximately 10 pg/mL in 20% fetal bovine serum for all infections tested.	785	None	[55]
To differentiate typhoid and DENV infections in human sera with confocal Raman microscope.	12 distinct bands were identified for typhoid infected samples (562, 649, 716, 780, 838, 1,099, 1,144, 1,156, 1,260, 1,386, 1,556, and 1746 cm^−1^) and 4 for DENV-infected samples (756, 1,218, 1,672, and 1,686 cm^−1^).	785	PCA-DA	[54]

DENV, Dengue virus; IgG, immunoglobulin G, IgM, immunoglobulin M; NS1, nonstructural protein 1; PCA-DA, principal component analysis-factorial discriminant analysis; PFU, plaque-forming unit; PLS, partial least squares; RS, Raman spectroscopy; RVFV, Rift Valley fever virus; SERS, surface-enhanced Raman spectroscopy; SVM, support vector machine; WNV, West Nile virus; ZIKV, Zika virus.

### Application of MIRS for the diagnosis of malaria parasites

Asexual stages of *P*. *falciparum* could be differentiated with Synchrotron Fourier transform infrared microspectroscopy and artificial neural network (ANN) based on the 2,800 to 3,100 cm^−1^ and to 1,000 to 1,800 cm^−1^ MIR regions with an accuracy of 100% for all stages tested (rings, trophozoites, and schizonts) [64]. Stages of *P*. *falciparum* in human O+ RBCs were differentiated with attenuated total reflectance Fourier transform infrared (ATR-FTIR) spectroscopy where rings, trophozoites, and gametocytes were distinct within 1,000 to 3,100 cm^−1^ regions. The study also identified that the limit of detection was <1 parasite/μL for the ring stage blood samples [65].

When MIRS was used for the detection of *P*. *falciparum* in human whole blood with parasitaemia ranging from 0% to 5%, a sensitivity of 70% and a specificity 98% was achieved [66]. Moreover, high-resolution infrared images of single cells infected with *P*. *falciparum*, changes in MIRS spectra region relating to increases in amide A band (consisting of N–H stretching modes of protein and C–H stretching region of lipids), and unsaturated fatty acids in infected cells could be identified [67]. Focal plane array-Fourier transform infrared (FPA-FTIR) imaging spectroscopy identified *P*. *falciparum* blood stages at a single-cell level [68] and a high-resolution FTIR could detect single malaria parasite-infected erythrocytes [67].

The effect of 3 different anticoagulants including sodium citrate, potassium ethylenediaminetetraacetic acid, and lithium heparin on plasma and whole blood in aqueous and dry phase on ATR-FTIR spectral signatures was tested. It was found that anticoagulants heavily influenced the spectra of dry blood samples compared to wet samples. Of the 3 anticoagulants tested, lithium heparin affected the mid-infrared spectra the least [69]. Findings from 2 most recent studies indicate MIRS can detect *P*. *falciparum* field-collected human blood spots on filter paper. Mwanga and colleagues identified *P*. *falciparum* with FTIR from field samples collected in Tanzania where an accuracy of 92%, sensitivity of 92.8%, and specificity of 91.7% in comparison to PCR findings was achieved [70]. The second study was done with *P*. *falciparum* samples collected in Thailand where a sensitivity of 92% and a specificity of 97% in comparison to PCR was observed [71]. A summary of the studies that applied MIR for diagnosis of malaria are indicated in Table 4, and an example of a MIR spectra for malaria infected and uninfected red blood cells is shown in Fig 2.

**Table 4:** A summary of articles reviewed that applied MIRS for diagnosis of malaria parasites.

Objective/s	Key finding/s	Wavenumber range/cm^−1^	Data processing technique	Reference
To distinguish asexual life cycle stages of *P*. *falciparum* in human RBCs with Synchrotron-FTIR microspectroscopy and ANN.	ANN analysis of the 1,000–1,800 cm^−1^ and 2,800–3,100 cm^−1^ spectral region differentiated infected from noninfected RBCs with a sensitivity of 100% and a specificity 92%. The regions also differentiated rings, trophozoites, and schizonts with 100% accuracy.	1,000–3,100	ANN	[64]
To differentiate and quantify early stage *P*. *falciparum* parasites in human RBCs with ATR-IR.	Best predictions were obtained from lipid C–H stretching at 2,800–3,100 cm^−1^. Ring, trophozoite, and gametocyte stages could be differentiated. The detection limit was 0.00001% parasitaemia.	600–4,000	PCA and PLS	[65]
To detect and differentiate *P*. *falciparum* stages in human RBCs with FPA-FTIR imaging spectroscopy.	Gametocyte and trophozoite haemozoin band were observed at 1,208 cm^−1^ compared to uninfected and ring stage RBCs. Other spectra peaks used to differentiate *P*. *falciparum* stages were in the 2,917 cm^−1^ and 2,955 cm^−1^ regions. Images of uninfected, ring, schizont, and trophozoite stages could be differentiated.	950–3,100	PCA	[68]
To diagnose *P*. *falciparum*-infected human RBCs at a single-cell level on a microscope slide with FPA-FTIR spectroscopy.	High-resolution infrared images of single cells infected with *P*. *falciparum* were taken and the distinction of the digestive vacuole was observed. A higher amount of amide A band and unsaturated fatty acids were observed in infected cells.	2,500–3,600	PLS-DA	[67]
To explore the effects of 3 anticoagulants (sodium citrate, potassium ethylenediaminetetraacetic acid, and lithium heparin) on aqueous and dry phase of human whole blood with ATR-FTIR spectroscopy.	Among the 3 anticoagulants tested, lithium heparin caused the least difference in the mid-infrared spectra. In wet blood samples, the anticoagulant influence on spectra was much less significant than dry samples.	650–3,900	PCA and PLS	[69]
To detect spiked *P*. *falciparum*, glucose, and urea in human whole blood with ATR-FTIR spectroscopy.	Sensitivity and specificity for detecting *P*. *falciparum* with parasitaemia >0.5% in whole blood was 70% and 98%, respectively.	700–3,000	PLS-regression and PLS-DA	[66]
To diagnose malaria infection in blood from malaria patients in Thailand with ATR-FTIR spectroscopy.	A sensitivity of 90% and specificity of 91% were achieved with PLS-DA and a sensitivity of 92% and specificity of 97% were achieved when SVM was used.	700–3,140	PLS-DA and SVM	[71]
To distinguish between malaria positive and negative dried blood spots contained in filter papers from malaria patients in Tanzania with ATR-FTIR spectroscopy relative to qPCR.	An accuracy of 92%, sensitivity of 92.8%, and specificity of 91.7% was achieved.	883–1,730	Logistic regression	[70]

ANN, artificial neural network; ATR-IR, attenuated total reflectance infrared; FPA, focal plane array; FTIR, Fourier transform infrared; MIRS, mid-infrared spectroscopy; PCA, principal component analysis; PLS, partial least squares; PLS-DA, partial least squares-discriminant analysis; qPCR, quantitative PCR; RBC, red blood cell; SVM, support vector machine.

### Application of MIRS for surveillance of malaria and arbovirus vectors

Highly variable accuracies were observed when MIRS was used to predict the age of laboratory-reared *Anopheles arabiensis* and *Anopheles gambiae* that ranged between 1 to 15 days old. Predictive accuracies of 15% to 97% and 10% to 100% were attained for *An*. *gambiae* and *An*. *arabiensis* mosquito species, respectively. Lower predictive accuracies were observed for middle age mosquitoes within 3 to 11 days old age group compared to 1 or 15 days old mosquitoes for both species [72]. The same technique also differentiated between field-collected *An*. *arabiensis* and *An*. *gambiae* with an accuracy of 82.6% [72]. A separate study used ATR-FTIR spectroscopy to predict the age of laboratory-reared *w*Mel-infected *Ae*. *aegypti* with an accuracy of 95% to 97% and to detect *Wolbachia* infections in *Ae*. *aegypti* field mosquitoes with an accuracy of 90% compared with that of PCR results. However, higher predictive accuracies of 95% to 97% were observed when mosquitoes were 2 and 10 days old. A significant difference in biochemical components between male and female was also identified by ATR-FTIR spectroscopy and the technique differentiated the 2 groups with a specificity and sensitivity of 95% to 100% [73].

Identification of the origin of a mosquito blood meal is crucial for the assessment of their host preference. Furthermore, upscaling MIRS into field studies will require models that are robust enough to accurately predict mosquitoes with various abdominal statuses including the source of their blood and blood digestion stages. MIRS has been used to successfully differentiate laboratory-reared *An*. *arabiensis* fed on goat, bovine, chicken, and human blood meals with predictive accuracies of 96%, 97%, 100%, and 100%, respectively [74]. A summary of the studies that applied MIR for surveillance of malaria and arbovirus vectors is shown in Table 5.

**Table 5:** A summary of articles reviewed that applied MIRS for surveillance of malaria and arbovirus vectors.

Objective/s	Key finding/s	Wavenumber range/cm^−1^	Data processing technique	Reference
To differentiate between age and species of *An*. *gambiae* and *An*. *arabiensis* with ATR-FTIR spectroscopy.	MIRS differentiated field-collected sugar-fed and gravid mosquitoes with an accuracy of 82.6%. Age prediction for *An*. *gambiae* ranged from 90%–95% for 1 and 15 days old and 10%–60% for 3, 5, 7, 9, and 11 days old. Age prediction for *An*. *arabiensis* ranged from 60%–100% for 1, 7, 9, and 11 days old and 5%–40% for 3, 5, and 15 days old mosquitoes.	400–4,000	Logistic regression	[72]
To distinguish between various vertebrate abdominal blood meals ingested by *An*. *arabiensis* with ATR-FTIR spectroscopy and supervised machine learning.	Accuracies of 97% (Bovine blood), 100% (Human blood), 96% (Goat blood), and 100% (Chicken blood) were achieved.	500–4,000	Logistic regression	[74]
To determine sex, age, and the presence of *Wolbachia* (*wM*el strain) in laboratory and field mosquitoes with ATR-FTIR spectroscopy.	Infection among laboratory-reared mosquitoes was predicted with >95% sensitivity and specificity. Mosquitoes were grouped into 2 or 10 days old age groups with an ROC of 0.991, and their sex was predicted with 97.4%. The infection status of field mosquitoes was predicted with a sensitivity of 84% and a specificity 92%, and their sex was differentiated with sensitivity and specificity ranging from 95%–100%.	700–3,600	PLS-DA	[73]

ATR, attenuated total reflectance; FTIR, Fourier transform infrared; MIRS, mid-infrared spectroscopy; PLS-DA, partial least squares-discriminant analysis; ROC, receiver operation characteristic.

### Application of Visible-NIRS for malaria and arbovirus vector surveillance

All studies on surveillance of mosquito vectors were conducted using a Labspec NIR spectrometer (Malvern Panalytical, Malvern, United Kingdom) whose wavenumber range is 4,000 to 28,571 cm^−1^. Most studies applied partial least squares (PLS) regression for data analysis to predict the age, infection, and species of mosquitoes of malaria and arbovirus-transmitting mosquitoes (Table 6).

**Table 6:** A summary of articles reviewed that applied Visible-NIRS for diagnosis and surveillance of malaria and arbovirus vectors. All studies on surveillance of mosquito vectors were conducted using a LabSpec NIR spectrometer (Malvern Panalytical, Malvern, UK).

Objective/s	Key finding/s	Wavenumber range/ cm^−1^	Data processing technique	Reference
To determine the effects of dietary status on NIRS age grading of laboratory-reared *Ae*. *aegypti*.	When all experimental groups were used in the model, the highest predictive accuracy of 90% was achieved.	4,255–14,286	PLS	[84]
To detect and differentiate 2 strains of laboratory-reared *Ae*. *aegypti* infected with *Wolbachia pipientis* (*w*MelPop and *w*Mel).	*w*MelPop infected and uninfected females and males were differentiated with 96% and 87.5% accuracy, respectively. *w*Mel infected and uninfected females and males were differentiated with an accuracy of 92% and 89%, respectively. *w*MelPop and *w*Mel infected females and males were differentiated with an accuracy of 96.6% and 84.5%, respectively.	4,255–14,286	PLS	[91]
To predict the age of laboratory-reared male and female wild-type and *Wolbachia*-infected *Ae*. *aegypti*.	The age of female *w*Mel and *w*MelPop-infected *Ae*. *aegypti* was predicted as <8 or ≥8 days old with 83% and 78% accuracy, respectively. The age of wild-type female *Ae*. *aegypti* for the same age group was predicted with 91% accuracy.	4,255–14,286	PLS	[81]
To predict the age of laboratory-reared *Ae*. *albopictus Skuse*.	When grouped into <7 or ≥7 days old age groups, an accuracy of 94.5% was achieved, and when grouped into >7, 7–13, and > 13 days old, an accuracy of 70.5% was achieved.	4,255–14,286	PLS	[82]
To detect ZIKV in heads/thoraces and abdomens of laboratory-reared *Ae*. *aegypti* mosquitoes.	The predictive accuracy was 97.3% when spectra from heads/thoraces were used. However, when spectra from abdomens were used, a predictive accuracy of 88% was observed relative to RT-qPCR findings.	4,255–14,286	PLS	[90]
To predict the age of *Ae*. *albopictus* adults reared from the laboratory and wild pupae.	Age prediction models for laboratory-reared *Ae*. *albopictus* differentiated 2- and 15-day-old mosquitoes but could not distinguish *Ae*. *albopictus* mosquitoes reared from wild pupae.	4,255–14,286	PLS and PCA	[83]
To determine age and species of *An*. *arabiensis* and *An*. *gambiae* from the lab and field.	Field-caught and laboratory samples of the 2 cryptic species were differentiated with approximately 80% and 100% accuracy, respectively. Age prediction accuracy into <7 and ≥7 days old age groups was approximately 80% for female mosquitoes. Age prediction accuracy for male mosquitoes >7 days old was ≥85% and for <7 days old was approximately 50%.	4,255–14,286	PLS	[75]
To differentiate and predict the age of *An*. *arabiensis* and *An*. *gambiae* s.s reared in a semi-field system.	Age prediction accuracy of *An*. *arabiensis* and *An*. *gambiae* into <7 or ≥7 days old age groups were at 89% and 78%, respectively. Species were differentiated with 89% accuracy. Wild-caught *An*. *gambiae* were identified with 90% accuracy.	4,255–14,286	PLS	[33]
To investigate the effects of various preservation methods (Carnoy, drierite, ethanol, refrigerated, and silica gel) on *An*. *gambiae* age prediction with NIRS.	Desiccants, refrigeration, and RNAlater were generally good preservation methods based on NIRS age prediction accuracies when compared to fresh samples (>80% accuracy) and 95% confidence interval of <1.2 days). For mosquitoes stored >50 days, Carnoy and silica gel had the closest accuracies of 81.7% and 81%, respectively, compared with that of fresh samples.	4,255–20,000	PLS and regression	[86]
To evaluate RNAlater as a preservative tool for *An*. *gambiae* and *An*. *arabiensis* when using NIRS for age and species grading.	Age prediction accuracy of mosquitoes into <7 or ≥7 days old age groups was 83% for fresh and 90% for preserved. For species identification, accuracies were 82% for fresh and 80% for RNAlater.	4,255–20,000	PLS	[85]
To predict the age of mixed species of *Anopheles* mosquitoes reared from wild larvae/pupae under varying environmental conditions and to determine whether exposure to pyrethroids affected their age prediction accuracy.	The age of wild larvae into <7 or ≥7 days old age groups was predicted with an accuracy of 79%. *Anopheles* mosquitoes that were not exposed, exposed, resistant, and susceptible, to pyrethroids were predicted with 79%, 79%, 82%, and 78% accuracies, respectively.	4,255–20,000	PLS	[76]
To evaluate the effect preservation methods on the predictive accuracy of NIRS for species differentiation of laboratory-reared *An*. *gambiae* s.s. and *An*. *arabiensis*.	Mosquitoes can be preserved in silica gel for up to 50 weeks with a predictive accuracy of 89.6% for *An*. *gambiae* and 90.4% for *An*. *arabiensis* when compared with fresh mosquitoes.	4,000–14,286	PLS	[87]
To develop regression and classification models for predicting parity status and *P*. *falciparum* sporozoites using wild and lab-reared mosquitos.	Age prediction accuracy of independent datasets varied from 41%–69.6% after PLS regression analysis was applied. Highest accuracy of predicting nulliparous from sporozoite positive mosquitoes was 62.5%.	4,255–20,000	PLS	[80]
To identify *P*. *berghei*-infected laboratory-reared *An*. *stephensi* mosquitoes.	Differentiated between infected and uninfected *An*. *stephensi* female mosquitoes with an accuracy of 72%, sensitivity of 70%, and specificity of 84%.	4,000–28,571	PLS	[88]
To identify differences between spectra of laboratory-reared and wild-caught *An*. *arabiensis* mosquitoes.	No significant differences were observed between spectral signatures of laboratory-reared and wild-type *An*. *arabiensis*. Silhouette coefficient of 0.25 indicated no clustering of data points based on the environment of the mosquitoes.	4,000–20,000	k-means, hierarchical cluster analyses, and PLS	[79]
To detect oocyst and sporozoite stage *P*. *falciparum* infection in laboratory-reared *An*. *gambiae*.	Oocyst stages were predicted with 87.7% accuracy and sporozoite stages were predicted with 94.5% accuracy when validated against qPCR findings. These predictive accuracies positively correlated with the parasite concentration	4,167–20,000	PLS	[89]
To determine if ANN instead of PLS regression age prediction models for *Anopheles* and *Aedes* improves the current accuracy of NIRS for mosquito age grading.	Compared to PLS, the root mean squared error was approximately 2% lower when ANN was used for age prediction. When regression models were interpreted as binary classifiers, ANN regression model accuracies improved by approximately 10% for both species. Generally, higher predictive accuracies were observed for ANN compared to PLS in independent test sets.	4,000–14,286	PLS and ANN	[77]
To predict the parity status of wild, *An*. *arabiensis* and *An*. *gambiae*, with the use of an autoencoder coupled with ANN models.	The prediction of accuracies of parity status were improved by 13%–21% depending on the source of mosquitoes and the sample size for *An*. *arabiensis* and *An*. *gambiae* with the use of autoencoder coupled with ANN models in comparison to ANN models alone.	4,000–20,000	ANN and autoencoder	[78]
To predict the time post death of ZIKV, CHIKV, and *Wolbachia* infection in female *Ae*. *aegypti* mosquitoes.	The prediction accuracy for fresh, 1 day old, 2–4 days old, and 5–7 days old achieved overall accuracies of 93.2%, 97%, and 90.3% for ZIKV, CHIKV, and *Wolbachia*, respectively, in dead *Ae*. *aegypti* female mosquitoes.	4,255–14,286	PLS	[92]
To identify *P*. *berghei* infection in mice whole blood.^a^	A peak at 650 nm was associated with *P*. *berghei* infection at 4 to 7 days post infection. (*P* = 0.1094).	200–1,200	None	[93]

^a^Diagnosis of malaria parasites study.

ANN, artificial neural network; CHIKV, Chikungunya virus; NIRS, near-infrared spectroscopy; PCA, principal component analysis; PLS, partial least squares; qPCR, quantitative PCR; RT-qPCR, quantitative reverse transcription PCR; ZIKV, Zika virus

In the past 12 years, NIRS has been used as an alternative strategy for age and species determination for malaria and arbovirus vectors in a range of studies under varying environmental conditions. Initially, NIRS’ potential for predicting the age and species was demonstrated on laboratory-reared mosquitoes where it was reported to be accurate for predicting the age of those mosquitoes into ±3 days of their actual age or into ≤ 7 days ≥ old age group and for differentiating *An*. *gambiae* from *An*. *arabiensis* with >90% accuracy [75]. Subsequent studies using *An*. *gambiae* and *An*. *arabiensis* reared in a semi-field system reported similar accuracies for age and species prediction [33,76]. More recent studies have indicated that the chronological age of *An*. *gambiae* and *An*. *arabiensis* can be improved using alternative machine learning techniques such as ANN as opposed to PLS regression analysis used in previous studies [77,78].

NIRS spectral features of laboratory-reared and wild-type *An*. *arabiensis* mosquitoes were shown to be identical regardless of the environment and diet of the mosquito (i.e., field-caught and laboratory-reared mosquitoes). Based on these findings, NIRS prediction models developed from laboratory-reared mosquitoes with known age could be possibly relied upon for predicting the age of wild mosquito populations with unknown age [79]. Two studies have demonstrated the potential of NIRS to predict the age of wild *An*. *arabiensis* and *An*. *gambiae* mosquitoes. Krajacich and colleagues [80] reported age prediction accuracy of 73.5% to 97% for wild and 69.6% for semi-field mosquitoes. This accuracy has recently been improved using an autoencoder and ANN to predict the parity status of field mosquitoes [78]. Findings from 2 other studies indicated that NIRS could differentiate field-caught *An*. *gambiae* and *An*. *arabiensis* with a predictive accuracy of 90% [33,75]. NIRS has also been used to predict the age of laboratory-reared *Ae*. *aegypti* [81] and *Ae*. *albopictus* [82] with or without *Wolbachia* with similar accuracies as those recorded for *An*. *gambiae* and *An*. *arabiensis*. However, when NIRS was used to predict *Ae*. *albopictus* mosquitoes reared from wild pupae using a model developed from laboratory-reared mosquitoes, young and old mosquitoes could not be differentiated [83]. Based on the authors’ description of their experimental design, the inability to predict the age of mosquitoes collected from wild pupae is most likely due to a weak predictive model that failed to capture the heterogeneity of the wild population including variation in the larval diet. Alternatively, the small sample size used for model development was not robust [83]. A previous study indicated that *Anopheles* mosquitoes reared from wild pupae could be predicted accurately if models were developed from a similar mosquito population and neither species type nor exposure to pyrethroids affected the ability of NIRS to predict their age [76]. However, larval and adult diets have been previously shown to have an influence on age-related spectral signatures [84].

Mosquitoes are commonly stored in various preservatives prior to spectral collection. Several studies have demonstrated the effect of a range of preservation techniques on the predictive accuracy of NIRS for age and species of *An*. *gambiae* and *An*. *arabiensis*. When RNAlater was used as a preservative for 1 to 3 weeks, the prediction accuracy was relatively higher (90%) for *An*. *gambiae* s.s and *An*. *arabiensis* compared to the accuracy of 83% for freshly scanned mosquitoes [85]. However, there appeared to be a decline in accuracy from 86% to 75.6% when mosquitoes were preserved for a longer period of time (50 to 62 days) [86]. Similarly, a light decrease (on average as 3.6%) in accuracy when differentiating *An*. *arabiensis from An*. *gambiae* were observed after 50 weeks of storage in silica gel [87]. For storage up to 4 weeks, RNAlater at 4°C, refrigeration at 4°C and silica gel are the recommended options for age and species prediction [86,87]. The fact that the prediction accuracy of samples stored in silica gel is comparable to the accuracy of fresh samples is encouraging as it means vector control programs would not be required to modify their current collection and storage protocols to adopt the NIRS technique. However, although silica gel is cost-effective, maintaining desiccation for a long period of time is a challenge. Due to its high cost, RNAlater is generally recommended for small-scale studies that require RNA extraction to stabilise RNA in the samples prior to analysis. Samples in RNAlater can stay at room temperature for a maximum of 2 weeks, hence RNAlater could be an alternative to silica gel for field work where access to a fridge is limited.

NIRS has been used to identify mosquitoes infected with various pathogens such as *P*. *berghei*, *P*. *falciparum*, *Wolbachia*, CHIKV, and ZIKV. A prediction accuracy of 72% was achieved when *P*. *berghei*-infected *Anopheles stephensi* were differentiated from uninfected mosquitoes [88]. *P*. *falciparum* was detected in laboratory-reared *An*. *gambiae*, with accuracies of 88% for oocyst stage and 95% for sporozoite stage (14 dpi). This predictive accuracy positively correlated with the concentration of the parasite within the mosquito [89]. ZIKV in laboratory-reared *Ae*. *aegypti* mosquitoes was predicted with an overall accuracy of 97.3% in the heads/thoraces and 88.8% in abdomens in comparison to RT-qPCR [90] and *w*Mel-infected *Ae*. *aegypti* mosquitoes were predicted with accuracies of 92% for female and 89% for male mosquitoes [91]. Similarly, the presence of *w*MelPop in *Ae*. *aegypti* females and males was predicted with accuracies of 96% and 87.5%, respectively [91]. Furthermore, NIRS could differentiate between *w*Mel and *w*MelPop-transinfected mosquitoes with predictive accuracies of 96.6% for females and 84.5% for males [91]. Overall, NIRS detected *Wolbachia* in females more accurately than in male mosquitoes and in *w*MelPop more accurately than *w*Mel probably based on concentration levels. Lastly, a recently published article provides evidence that NIRS can also detect the presence of *Wolbachia*, ZIKV, and Chikungunya viruses in mosquitoes 7 days post their death [92]. A summary of the studies that applied NIRS for surveillance of malaria and arbovirus vectors is shown in Table 6, and an example of NIRS raw spectra of ZIKV infected and uninfected female *Ae*. *aegypti* is shown in Fig 1.

### Application of Visible-NIRS for diagnosis of malaria parasites

Only one study has applied NIRS to detect *P*. *berghei* in the whole blood of mice infected with rodent malaria. The study compared NIRS spectra of 6 *P*. *berghei*-infected mice and 6 uninfected mice. A characteristic peak at 650 nm related to *P*. *berghei* infection increased in intensity with rising parasitaemia (R^2^ value = 0.68) [93]. The study referenced has been added to Table 6.

### Knowledge gaps in the application of Raman and infrared spectroscopy for malaria and arboviruses

Although several studies have demonstrated the potential of RS for diagnosis of both malaria and arboviruses in laboratory settings (Table 2), the validation of RS under real-world conditions is an area that has not been fully investigated. Moreover, no studies were identified that have assessed the potential of RS for surveillance and characterisation of mosquito vectors. The use of MIRS for diagnosis of malaria has been recently demonstrated in the laboratory by several studies and in the field by 2 studies [70,71], whereas its application as surveillance tool for malaria vectors has only been demonstrated by 3 studies in the laboratory [72–74]. Future research should assess the potential of MIRS for the diagnosis of arboviruses in humans and validate its feasibility under field conditions for both malaria and arboviruses. NIRS has been used in several studies for the surveillance and characterisation of mosquito vectors into age groups, species identity, and infection status. However, most of the studies were conducted on laboratory samples. Only 2 studies reported that NIRS can differentiate field-collected *An*. *gambiae* from *An*. *arabiensis* [33,75] and parous from nulliparous malaria vectors [78,80]. Further work is required to demonstrate the full potential of NIRS in the field and to validate it against gold standard techniques. Finally, only 1 study demonstrated that NIRS can detect *Plasmodium* in mice blood [93] opening an opportunity to investigate its diagnostic capacity for malaria and arboviruses in human tissues.

## Conclusions

The objective of this systematic review was to demonstrate the various studies that have used RS, MIRS, and NIRS as diagnostic tools for malaria and arboviruses or as surveillance tools for mosquito vectors. These spectroscopy techniques are rapid, and NIRS and RS for example can be applied non-invasively without consuming reagents. Their application for the diagnosis or surveillance of malaria and arboviruses is a relatively new area of research. This review has identified opportunities which could potentially assist in the development of these techniques as future cost-effective, point-of-care diagnostics, or rapid surveillance tools for mosquito vectors. For example, the recent development of multiple infrared-based and Raman devices paired with advances in machine learning could revolutionise the application of these techniques and subsequently enable their real-time application in the field. Of importance is the standardisation and optimisation of currently available infrared and Raman spectra collection techniques to enable reproducibility between samples and instruments. Also required is a comprehensive assessment of Raman and infrared spectroscopy techniques to determine their utility in the diagnosis of infections that cause similar symptoms in humans such as arboviruses. Finally, comparative studies to determine the relationship between spectral signatures for mosquitoes infected with various pathogens including those that carry resistance genes should be investigated. Other factors that need to be investigated include how host immunity, age, gender, and blood type may all have an influence on spectral signatures collected.

The assessment of these devices in the field should be prioritised with the aim of developing point-of-care tools to support epidemiological studies of malaria and arboviruses and ultimately aide in combating current and future outbreaks of these infectious diseases. User-friendly protocols coupled with field deployable devices and cloud-based artificial intelligence platforms would improve the speed and reduce the cost of current disease surveillance programs by several magnitudes to facilitate rapid decision making by policy makers.

**Key learning points**

- Research in the application of RS as a potential surveillance tool for mosquito vectors is recommended.
- Research in the application of MIRS as a potential tool for diagnosis of arboviruses is recommended.
- Research in the application of NIRS for diagnosis of malaria and arboviruses is recommended.
- A protocol for standardisation of sample and spectra collection is required to harmonise the application of various spectroscopy techniques in multiple settings.

**Top five papers**

1. Hobro AJ, Konishi A, Coban C, Smith NI. Raman spectroscopic analysis of malaria disease progression via blood and plasma samples. Analyst. 2013;138(14):3927–33.
2. Mwanga EP, Minja EG, Mrimi E, Jiménez MG, Swai JK, Abbasi S, et al. Detection of malaria parasites in dried human blood spots using mid-infrared spectroscopy and logistic regression analysis. Malar J. 2019;18(1):341.
3. Heraud P, Chatchawal P, Wongwattanakul M, Tippayawat P, Doerig C, Jearanaikoon P, et al. Infrared spectroscopy coupled to cloud-based data management as a tool to diagnose malaria: a pilot study in a malaria-endemic country. Malar J. 2019;18(1):348.
4. Milali MP, Kiware SS, Govella NJ, Okumu F, Bansal N, Bozdag S, et al. An autoencoder and artificial neural network-based method to estimate parity status of wild mosquitoes from near-infrared spectra. PLoS ONE. 2020;15(6):e0234557.
5. Santos LMB, Mutsaers M, Garcia GA, David MR, Pavan MG, Petersen MT, et al. High throughput estimates of *Wolbachia*, Zika and chikungunya infection in *Aedes aegypti* by near-infrared spectroscopy to improve arbovirus surveillance. Commun Biol. 2021;4(1):67.

## Supporting information

S1 Table PRISMA 2009 checklist. (DOC)

S2 Table Summary of excluded full text articles. (DOCX)
