# Supplementary material for: The application of spectroscopy techniques for diagnosis of malaria parasites and arboviruses and surveillance of mosquito vectors: A systematic review and critical appraisal of evidence
Source: PLoS Negl Trop Dis. 2021 Apr 22;15(4):e0009218. doi: 10.1371/journal.pntd.0009218 (PMC8061870; doi:10.1371/journal.pntd.0009218)
Supplement: S2 Table — (DOCX) [file pntd.0009218.s002.docx]

| Article |  | Summary of article | Reason for exclusion |
| --- | --- | --- | --- |
| Puntharod, Ratchadaporn, Grant T. Webster, Mehdi Asghari-Khiavi, Keith R. Bambery, Feryal Safinejad, Shadi Rivadehi, Steven J. Langford, Kenneth J. Haller, and Bayden R. Wood. "Supramolecular interactions playing an integral role in the near-infrared Raman “excitonic” enhancement observed in β-hematin (malaria pigment) and other related heme derivatives." *The Journal of Physical Chemistry B* 114, no. 37 (2010): 12104-12115. | | Monitoring the interactions of heme groups with Resonance Raman spectroscopy. | Irrelevant to diagnosis or surveillance of malaria. |
| Christensen, Dale, Anja Rüther, Kamila Kochan, David Pérez-Guaita, and Bayden Wood. "Whole-organism analysis by vibrational spectroscopy." Annual Review of Analytical Chemistry 12 (2019): 89-108. | | Discussion of new and recent infrared and RS used in whole organism analysis. | Literature review article. |
| Boyer, Chantal, Karen Gaudin, Tina Kauss, Alexandra Gaubert, Abdelhakim Boudis, Justine Verschelden, Mickaël Franc et al. "Development of NIRS method for quality control of drug combination artesunate–azithromycin for the treatment of severe malaria." Journal of pharmaceutical and biomedical analysis 67 (2012): 10-15. | | Development of NIRS methods for the analysis of antimalarial antibiotic  in gelatin capsule. | Irrelevant to diagnosis or surveillance of malaria. |
| Selvan, Sekaran Muthamil, Kabali Vijai Anand, Kasivelu Govindaraju, Selvaraj Tamilselvan, Vijayakumar Ganesh Kumar, Kizhaeral Sevathapandian Subramanian, Malaisamy Kannan, and Kalimuthu Raja. "Green synthesis of copper oxide nanoparticles and mosquito larvicidal activity against dengue, zika and chikungunya causing vector Aedes aegypti." *IET nanobiotechnology* 12, no. 8 (2018): 1042-1046. | | *Tridax procumbens* leaf extract synthesized copper oxide nanoparticles were used to counter larvicidal activity of *Aedes aegypti.* | NIRS was used to characterise copper oxide nanoparticles.  Irrelevant to diagnosis or surveillance of malaria. |
| Li, Zihan, Luca Leustean, Fatih Inci, Min Zheng, Utkan Demirci, and Shuqi Wang. "Plasmonic-based platforms for diagnosis of infectious diseases at the point-of-care." Biotechnology advances 37, no. 8 (2019): 107440. | | A comprehensive review on new and recent plasmonic-based biosensors for point of care infectious disease diagnostics. | Literature review article. |
| Kitahama, Yasutaka, and Yukihiro Ozaki. "Surface-enhanced resonance Raman scattering of hemoproteins and those in complicated biological systems." *Analyst* 141, no. 17 (2016): 5020-5036. | | A review article on SERS studies on hemeproteins on different metal substrates. | Literature review article. |
| Madzorera, Tatenda, Mthokozisi Sibanda, Walter Focke, Moshawe Madito, and Ncholu Manyala. "Malathion-filled trilayer polyolefin film for malaria vector control." Materials Science and Engineering: C 96 (2019): 419-425. | | Investigation of tri-layer films as potential ceiling or wall linings to prevent mosquito intrusion. | Micro-RS was used to determine tri-layer film structure.  Irrelevant to diagnosis or surveillance of malaria. |
| Visser, Benjamin J., Sophia G. de Vries, Emmanuel B. Bache, Janneke Meerveld-Gerrits, Daniëlle Kroon, Jimmy Boersma, Selidji T. Agnandji, Michèle van Vugt, and Martin P. Grobusch. "The diagnostic accuracy of the hand-held Raman spectrometer for the identification of anti-malarial drugs." Malaria journal 15, no. 1 (2016): 1-12. | | Determination of the diagnostic accuracy of a potable RS, to identify anti-malarial drugs. | Irrelevant to diagnosis or surveillance of malaria. |
| Versiani, Alice F., Ruiz G. Astigarraga, Eliseu SO Rocha, Ana Paula M. Barboza, Erna G. Kroon, Milene A. Rachid, Daniele G. Souza et al. "Multi-walled carbon nanotubes functionalized with recombinant Dengue virus 3 envelope proteins induce significant and specific immune responses in mice." Journal of nanobiotechnology 15, no. 1 (2017): 1-13. | | Evaluation of a novel alternative candidate vaccine against DENV3. | RS was used to characterize immunogens during vaccine development.  Irrelevant to diagnosis or surveillance of arboviruses. |
